# Bi-Phenotypic Trait May Be Conferred by Multiple Alleles in a Germplasm Population

**DOI:** 10.3389/fgene.2020.00559

**Published:** 2020-06-03

**Authors:** Fangdong Liu, Jianbo He, Wubin Wang, Guangnan Xing, Junyi Gai

**Affiliations:** ^1^Soybean Research Institute, Nanjing Agricultural University, Nanjing, China; ^2^MARA National Center for Soybean Improvement, Nanjing Agricultural University, Nanjing, China; ^3^MARA Key Laboratory of Biology and Genetic Improvement of Soybean (General), Nanjing Agricultural University, Nanjing, China; ^4^State Key Laboratory for Crop Genetics and Germplasm Enhancement, Nanjing Agricultural University, Nanjing, China; ^5^Jiangsu Collaborative Innovation Center for Modern Crop Production, Nanjing Agricultural University, Nanjing, China

**Keywords:** annual wild soybean (*G. soja* Sieb. and Zucc.), cultivated soybean [*G. max* (L.) Merr.], bi-phenotypic trait, χ2 association mapping, RTM-GWAS, Mendelian inheritance, marker-haplotype, gene-allele

## Abstract

The Chinese soybean germplasm pool (CSGP) comprises annual wild (WA), farmers’ landrace (LR) and released cultivar (RC) populations, and ecoregion subpopulations in WA/LR/RC (ecoregion IV/III/II/I). A representative sample consisted of 1,024 accessions was studied for pubescence color (PC) and flower color (FC). In the evolution from WA (brown PC and mainly purple FC) to LR then to RC, with above wild characteristic changed, while gray PC, and white FC emerged and their frequency increased. Using 36,952 genomic SNPLDB markers with 100,092 haplotypes, the association between markers and bi-phenotypic traits was detected using χ^2^ association analysis under single locus model and RTM-GWAS procedure under multi-locus model, respectively. Multiple markers co-associated with individual bi-phenotypic trait with the most significant markers containing multiple rather than two haplotypes even for a bi-phenotypic trait. On a marker/locus, each haplotype corresponds to two colors, except one (*FC-1-5*) out of 11 haplotypes for single color. The major candidate gene was annotated with its alleles identified from the population sequencing data. Similarly, multiple alleles identified and each corresponds to two colors except three (*a8*/*a9*/*b3*) out of 12 alleles for single color. The major haplotypes/alleles in LR and RC were traced to WA ecoregion subpopulations, the WA_*IV*_ and WA_*III*_ genotypes showed genetically more close to the cultivated subpopulations, therefore, WA from Ecoregion IV and III were inferred as the common ancestor for cultivated soybeans. The marker-haplotypes/gene-alleles not exactly coincided with the bi-phenotypic trait has challenged to the traditional Mendelian genetics, which was discussed and to be further studied.

## Introduction

Mendelian inheritance started the modern genetics using a pair of pea parents with a pair of relative characteristics (white vs. purple flower). The Mendelian genetics holds one-to-one correspondence relationship between genotype and phenotype. In soybean, since the previous genetic studies on pigmentation traits mainly used bi-parental populations, there were only two types of alleles involved and reported, while sometimes another gene/locus reported using another pair of parents, such as *T*/*t* and *Td*/*td* for pubescence color (PC), and *W1*/*w1*, *W3*/*w3*, *W4*/*w4*, *Wm*/*wm*, and *Wp*/*wp* for flower color (FC; Palmer et al., 2004). Therefore, simple Mendelian inheritance was found for the two traits as indicated above.

It is known that cultivated soybean [*G. max* (L.) Merr.] was domesticated from annual wild soybean (*G. soja* Sieb. and Zucc.) about 5,000 years ago (Zhou et al., 2015), originally annual wild soybean was with brown PC and purple FC (Zhuang et al., 1996), while in the cultivated soybeans, gray PC and white FC emerged, and with their frequencies increased gradually (Chang, 1990). Therefore, PC and FC were a bi-phenotypic trait in natural populations as indicated above. It is generally understood that anthocyanin accumulation determines PC and FC, flavonoid 3′5′-hydroxylase (*F3′5′H*), dihydroflavonol-4-reductase (*DFR*), and flavonoid 3′ hydroxylase (*F3′H*) play a crucial role in producing anthocyanin (Yang et al., 2010; Sundaramoorthy et al., 2017).

In natural population, the associated markers/loci for PC and FC have been reported, they may be different in positions on chromosomes, but generally similar, for instance, the loci associated with PC and FC were located at 18.5–18.7 Mb in Gm06, and 3.5–4.5 Mb in Gm13, respectively, (Sonah et al., 2015; Wen et al., 2015; Zhou et al., 2015; Han et al., 2016; Fang et al., 2017). However, a 2-haplotype SNP marker does not provide multiple haplotypes for the bi-phenotypic traits, and may not reflect the complex genetic basis of a bi-phenotypic trait in natural populations. To understand the genetic control of these bi-phenotypic traits, markers with multiple haplotypes should be used in association studies.

To find the associated markers for a qualitative trait (frequency data), the χ^2^ association analysis for individual markers under single-locus model in a contingency table was usually used. He et al. (2017) established a novel restricted two-stage multi-locus multi-allele genome-wide association study (RTM-GWAS) using a genomic marker called SNPLDB (SNP linkage disequilibrium block), characterized with multiple haplotypes to identify multiple alleles under a multiple-locus model for a quantitative or qualitative trait. Here, the tightly linked SNPs on genome are grouped into a SNPLDB which usually with multiple haplotypes due to multiple SNPs or with only two haplotypes if only a single SNP or several completely linked SNPs involved.

The present study aimed at to identify the genetic structure of the two simple but distinguishable bi-phenotypic traits (PC and FC) in soybeans based on associated marker and candidate gene analysis and to explore the evolutionary genetic relationship among the geographic ecoregion subpopulations. A large population composed of 1,024 accessions were collected and studied, as a representative sample in China. Both χ^2^ association analysis and RTM-GWAS procedure were used in association analysis for mutual checks. We found that a bi-phenotypic trait may be conferred by multiple haplotypes/alleles and one haplotype/allele may express different phenotypes in a germplasm population. It is a challenge for the traditional Mendelian inheritance, and we would report our findings for a public discussion.

## Materials and Methods

### Plant Materials and Field Experiment

The Chinese soybean germplasm pool (CSGP) is composed of mainly three populations, annual wild population (WA), farmers’ landrace population (LR), and released cultivar population (RC). A representative sample consisted of 1,024 accessions collected from the whole country, including 182 WA, 396 LR, and 446 RC, was provided by the National Center for Soybean Improvement. These accessions have their origination in the four soybean ecoregion subpopulations in China, i.e., I. Northern Single Cropping, Spring Planting Ecoregion; II: Huang-Huai-Hai Double Cropping, Spring and Summer Planting Ecoregion; III: Middle and Lower Changjiang Valley Double Cropping, Spring and Summer Planting Ecoregion; and IV: South and Southwest China Multiple Cropping Multiple Planting Ecoregion (Table 1 and Supplementary Figure S1). Here ecoregion IV is equivalent to the total of IV + V + VI in Wang and Gai’s (2002) ecoregion system for simplicity in the present study, e.g., WA_*IV*_: a subpopulation for annual WA from South and Southwest China Multiple Cropping Multiple Planting Ecoregion.

**TABLE 1:** The frequency distribution of bi-phenotypic traits in populations as well as ecoregion subpopulations in CSGP.

Population/	PC(%)		FC(%)	
Ecoregion subpopulation	Brown	Gray	Purple	White
WA(182)^*a*^	100.0	−	94.5	5.5
WA_*I*_(55)	100.0	−	96.4	3.6
WA_*II*_(59)	100.0	−	93.2	6.8
WA_*III*_(44)	100.0	−	90.9	9.1
WA_*IV*_(24)	100.0	−	100.0	−
LR(396)	63.1	36.9	66.4	33.6
LR_*I*_(45)	51.1	48.9	73.3	26.7
LR_*II*_(110)	51.8	48.2	50.9	49.1
LR_*III*_(78)	66.7	33.3	75.6	24.4
LR_*IV*_(163)	72.4	27.6	70.6	29.4
RC(446)	27.4	72.6	52.2	47.8
RC_*I*_(132)	7.6	92.4	44.7	55.3
RC_*II*_(183)	34.4	65.6	58.5	41.5
RC_*III*_(62)	30.6	69.4	46.8	53.2
RC_*IV*_(69)	43.5	56.5	55.1	44.9
CSGP(1024)	54.1	45.9	65.2	34.8

*PC, pubescence color; FC, flower color; WA, annual wild population; LR, farmers’ landrace population; RC, released cultivar population; and CSGP: the Chinese soybean germplasm pool. I: Northern Single Cropping, Spring Planting Ecoregion; II: Huang-Huai-Hai Double Cropping, Spring and Summer Planting Ecoregion; III: Middle and Lower Changjiang Valley Double Cropping, Spring and Summer Planting Ecoregion; and IV: South and Southwest China Multiple Cropping Multiple Planting Ecoregion. ^*a*^The number in parentheses is the accessions number of population or ecoregion subpopulation.*

The accessions were tested at Jiangpu Experimental Station of Nanjing Agricultural University, Nanjing, China (32°07′N, 118°62′E). Bi-phenotypic traits investigated are PC (gray vs. brown) and FC (white vs. purple).

### SNP Genotyping and SNPLDB Assembly

The CSGP was genotyped with RAD-seq (restriction site-associated DNA sequencing) at Beijing Genomics Institute (BGI), Shenzhen, China as described in the previous study (He et al., 2017). The resulted SNPs with missing rate > 20%, heterozygous rate > 20%, and minor haplotype/allele frequency < 1% were excluded, and a total of 145,558 high quality SNPs were obtained and used in the present study. Missing genotype were imputed using the software fastPHASE (Paul and Matthew, 2006). Then the SNP(s) within a linkage disequilibrium block were organized into a SNPLDB marker (*D*′ > 0.70) with haplotypes as its alleles. A single SNP isolated from LD blocks was also treated as a SNPLDB. If the haplotype/allele frequency was less than 1%, the haplotype was replaced with an approximate haplotype with a highest frequency. Finally, a total of 145,558 SNPs and 36,952 SNPLDBs with their 100,092 haplotypes (each SNPLDB with 2–14 haplotypes) were identified in CSGP (refer to Supplementary Data Sheet S1 for the phenotype and genotype data of pubescence color and flower color).

### Association Analysis Between SNPLDBs and Bi-Phenotypic Traits as Well as Candidate Genes

The single-locus model χ^2^ association analysis was conducted for individual SNPLDBs. The associated SNPLDBs with top 10 largest -log_10_*p* values were selected. At the same time, RTM-GWAS under multi-locus model (He et al., 2017) was also carried out for mutual checks with the single-locus model χ^2^ association analysis. The selected SNPLDBs/loci were annotated for their candidate genes, then the annotated genes (version Wm82. a1.v1.1) were retrieved from SoyBase^1^. The selected SNPLDBs were annotated for their possible candidate genes: at first, the candidate genes in the marker region with a 100-Kb flanking expansion were annotated; to identify the candidate gene from the annotated genes, the χ^2^ analysis was used to test the association between the SNPs in detected SNPLDBs and SNPs in the annotated genes, the significance level was set as 0.05 for each SNP in the annotated genes (Meng et al., 2016; Li et al., 2017). Based on the identified SNPLDBs and candidate genes, the frequency of marker-haplotypes and gene-alleles were analyzed for the three populations (WA, LR, and RC), and all ecoregion subpopulations (ecoregion IV, III, II, and I).

## Results

### Phenotypic Frequency Distribution of the Two Bi-Phenotypic Traits in Populations and Ecoregion Subpopulations

Bi-phenotypic traits are significantly changed from WA to LR and then to RC. Brown PC was primitive phenotype, its frequency showed a decrease tendency from WA (100.0%) to LR (63.1%) and then to RC (27.4%), while gray PC showed an increased tendency (Table 1). All WA subpopulations are brown PC; in LR, from LR_*IV*_ northward to LR_*I*_, the brown PC frequency showed a reduced tendency and vice versa for gray PC; in RC, the brown PC frequency was less than gray PC frequency in all subpopulations; brown PC as the primitive phenotype has the highest frequency in LR_*IV*_ of LR and RC_*IV*_ in RC, gray PC as a new phenotype increased gradually during the evolution (Table 1).

For FC, there showed purple FC frequency reduced tendency from WA (94.5%) to LR (66.4%) and then to RC (52.2%), and white FC showed an increased tendency (Table 1). In each subpopulation of WA, purple FC was the dominant part, the phenotype in WA_*IV*_ was purple but white FC already came out starting from WA_*III*_ and northward (Table 1). It should be explained here that the primitive FC for WA should be purple only since the most primitive ecoregion WA_*IV*_ performed only purple FC, it is because the annual wild soybean originated from perennial wild soybean in the low latitudes (north of 24°N, such as south China) and then disseminated to northern latitudes in East Asia (Xu et al., 1987; Hui et al., 1997; Kollipara et al., 1997). Thus WA_*IV*_ kept the most primitive FC constitution. In all subpopulations of LR, the purple FC frequency was more than white FC frequency; in RC, the purple and white FC frequency was about similar in each subpopulation (Table 1).

It seems that from WA to LR, new phenotype emerged and the bi-phenotypic traits formed. This change was enhanced in RC, while among the ecoregion subpopulations, those southern subpopulations (ecoregion IV of WA and LR) are more primitive in comparison to the northern subpopulations (ecoregion III, II, and I of WA and LR).

### Characteristics of Marker-Haplotypes and Corresponding Candidate Gene-Alleles Associated to the Two Bi-Phenotypic Traits in CSGP

**FIGURE 1:**
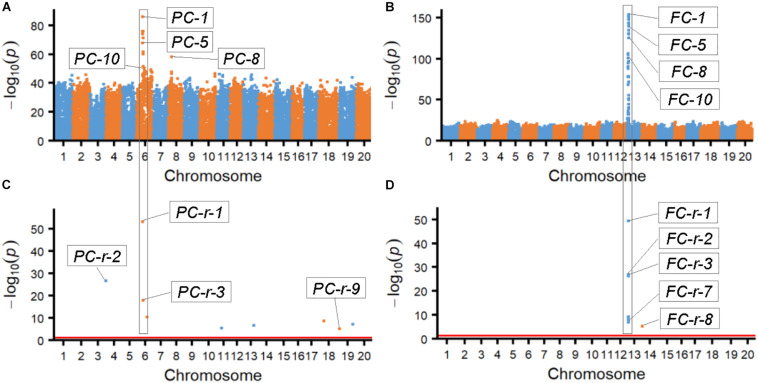
Manhattan figures of the two bi-phenotypic traits obtained from χ^2^ association analysis and RTM-GWAS procedure. **(A)** Manhattan map of pubescence color by χ^2^ association analysis. **(B)** Manhattan map of flower color by χ^2^ association analysis. **(C)** Manhattan map of pubescence color by RTM-GWAS. The *p*-values of association test in -log_10_ scale; *p*-values less than 1e-50 were randomly changed to a value between 1e-50 and 1e-55. **(D)** Manhattan map of flower color by RTM-GWAS. The *p*-values of association test in -log_10_ scale; *p*-values less than 1e-50 were randomly changed to a value between 1e-50 and 1e-55.

The results from association analyses showed that the SNPLDBs associated with the two bi-phenotypic traits were those with multi-haplotypes mostly rather than those with only bi-haplotypes and very few cases of one haplotype corresponding to a single phenotype. According to the probability value (-log_10_*p*) of the associated SNPLDBs, the most possible ones were chosen by χ^2^ association analysis (ten SNPLDBs for each trait; Figures 1A,B and Supplementary Table S1), the most possible locus for PC and FC were *PC-1* and *FC-1*, respectively, and the other associated loci with high significance located closely and associated to *PC-1* and *FC-1*, these closely related loci in fact were co-associated with *PC-1* and *FC-1*, respectively (Supplementary Table S1). On the other hand, among the nine and eight markers identified from RTM-GWAS (Figures 1C,D), the most possible SNPLDBs were *PC-r-1* and *FC-r-1*, with their highest contribution of 25.3% and 69.1% to phenotypic variance, respectively; interestingly, *PC-1* and *PC-r-1*, and *FC-1* and *FC-r-1* were in fact the same locus, respectively (Supplementary Table S1). It is curious that these most fitted SNPLDBs all contain multiple haplotypes, six haplotypes for *PC-1* and five haplotypes for *FC-1* (Supplementary Table S1), rather than two haplotypes even the 2-haplotype SNPLDBs closely located around the most fitted multi-haplotype SNPLDBs for the bi-phenotypic traits (Supplementary Tables S2, S3). It indicates that for these bi-phenotypic traits, not necessarily only two haplotypes involved, new haplotypes might happen in the germplasm population in the history.

There were six haplotypes on *PC-1*, but only five haplotypes in WA, all associated with brown PC with *PC-1-2* absent which emerged in LR; however, all the six haplotypes associated with both gray and brown PC in LR and RC. Similarly, there were five haplotypes on *FC-1*, only *FC-1-5* associated with a single purple FC but excluded in RC, the other four haplotypes associated with both white and purple FC (Table 2). This result indicates that most haplotypes expressed two phenotypes, a haplotype is not necessarily and completely corresponding to a single phenotype, in contrary, a phenotype is not necessarily and completely corresponding to a single haplotype, thus very few cases (only *FC-1-5*) of one haplotype corresponding to a single phenotype. This knowledge is completely different from the traditional understanding on Mendelian genetics that phenotypes and haplotypes are one to one corresponded. Since a marker may involve several genes, to know the exact gene-allele vs. phenotype relationship, the candidate gene conferring the bi-phenotypic trait and its alleles should be further examined.

**TABLE 2:** Phenotypic frequencies corresponding to the associated marker-haplotypes/gene-alleles for the two bi-phenotypic traits in CSGP (%).

Locus/gene	Haplotype/	WA		LR		RC	
	Allele	Brown	Gray	Brown	Gray	Brown	Gray
*PC-1*	*PC-1-1*^*a*^	1(100.0)^*b*^	−	107 (63.7)	61 (36.3)	70 (41.4)	99 (58.6)
	*PC-1-2*	−	−	11 (23.4)	36 (76.6)	7 (3.2)	212 (96.8)
	*PC-1-3*	107 (100.0)	−	66 (80.5)	16 (19.5)	4 (80.0)	1 (20.0)
	*PC-1-4*	32 (100.0)	−	37 (64.9)	20 (35.1)	36 (87.8)	5 (12.2)
	*PC-1-5*	29 (100.0)	−	23 (63.9)	13 (36.1)	4 (44.4)	5 (55.6)
	*PC-1-6*	13 (100.0)	−	6 (100.0)	−	1 (33.3)	2 (66.7)
*Glyma06g21551*	*a1*.CTATA^*a*^	145(100.0)^*b*^	−	204 (66.2)	104 (33.8)	112 (50.7)	109 (49.3)
	*a2*.TCAAC	12 (100.0)	−	17 (32.7)	35 (67.3)	7 (3.2)	211 (96.8)
	*a3*.CTTTA	21 (100.0)	−	26 (83.9)	5 (16.1)	2 (100.0)	−
	*a4*.CCAAC	2 (100.0)	−	2 (100.0)	−	−	2 (100.0)
	*a5*.CCATA	1 (100.0)	−	−	1 (100.0)	−	−
	*a6*.TCAAA	1 (100.0)	−	−	−	−	1 (100.0)
	*a7*.TCATA	−	−	−	−	1 (50.0)	1 (50.0)
	*a8*.CTAAC	−	−	1 (100.0)	−	−	−
	*a9*.TTAAC	−	−	−	1 (100.0)	−	−

**Locus/gene**	**Haplotype/**	**WA**		**LR**		**RC**	
	**Allele**	**Purple**	**White**	**Purple**	**White**	**Purple**	**White**

*FC- 1*	*FC-1-1*	11 (91.7)	1 (8.3)	228 (92.3)	19 (7.7)	205 (95.3)	10 (4.4)
	*FC-1-2*	−	5 (100.0)	13 (10.3)	113 (89.7)	27 (11.7)	203 (88.3)
	*FC-1-3*	63 (100.0)	−	6 (85.7)	1 (14.3)	1 (100.0)	−
	*FC-1-4*	44 (91.7)	4 (8.3)	13 (100.0)	−	−	−
	*FC-1-5*	54 (100.0)	−	3 (100.0)	−	−	−
*Glyma13g04210*	*b1*.GG	151 (93.8)	10 (6.2)	116 (47.7)	127 (52.3)	140 (39.9)	211 (60.1)
	*b2*.AG	10 (100.0)	−	146 (96.1)	6 (3.9)	93 (97.9)	2 (2.1)
	*b3*.GA	11 (100.0)	−	1 (100.0)	−	−	−

*PC, pubescence color; FC, flower color; WA, annual wild population; LR, farmers’ landrace population; RC, released cultivar population; and CSGP: the Chinese soybean germplasm pool. ^*a*^The first haplotype or allele. ^*b*^The number in parentheses is the percentage of accessions with the phenotype for the haplotype/allele.*

The results from candidate gene-allele analysis showed that the candidate genes associated to the two bi-phenotypic traits were those with multi-alleles mostly rather than those with only bi-alleles and very few cases of one allele corresponding to a single phenotype. To find the candidate genes in or around the associated SNPLDBs (±100 Kb), the genes were annotated based on the soybean genetics and genomics database from *G. max* (version Wm82.a1.v1.1) in SoyBase^1^. (i) In *PC-1* ± 100 Kb, *Glyma06g21551* was recognized as involved in the regulation of PC, and the five SNPs in this gene were tested for their significant association to the *PC-1* (*p* < 0.05) and nine candidate gene alleles were identified in CSGP (Supplementary Table S4). (ii) In *FC-1* ± 100 Kb, *Glyma13g04210* was recognized as involved in the regulation of FC, two SNPs were included (*p* < 0.05) and three candidate gene alleles were identified in CSGP (Supplementary Table S4). The identified candidate genes were all with function related to pigmentation, but they were different from the genes identified by Han et al. (2016) and Fang et al. (2017).

Like the associated SNPLDBs, these candidate genes all contains multiple alleles, nine alleles for *Glyma06g21551*, and three alleles for *Glyma13g04210*, rather than two alleles. For *Glyma06g21551*, six of the nine alleles, *a1-a6* in WA were with a phenotype of brown PC; *a1-a4* in LR and RC were with both brown and gray PC; *a7-a9* were new alleles in cultivated soybeans with *a8* for brown PC, *a9* for gray PC and *a7* for both PC colors (Table 2). For *Glyma13g04210*, *b1* was with both purple and white FC in WA, LR and RC, *b2* was with purple FC in WA but with both purple and white FC in LR and RC, and *b3* was the one with only purple FC (Table 2). Thus, only few cases (*a8*, *a9*, and *b3*) of one allele corresponding to a single color happened in CSGP.

From the above, multiple haplotypes/alleles conferring a same phenotype and one haplotype/allele conferring two phenotypes in a bi-phenotypic trait are popular phenomena in germplasm population. The two bi-phenotypic traits in wild soybeans are brown PC and mainly purple FC. During the process from wild to cultivated soybean, the gray PC and white FC emerged and increased after domestication (Chang, 1990; Zhuang et al., 1996). In the present study, the large sample of 1,024 was analyzed for association between the two bi-phenotypic traits and 36,952 SNPLDBs (each with 2–14 haplotypes, 2-haplotype markers accounting for 74.2%) using both χ^2^ association analysis and RTM-GWAS, both bi-phenotypic traits fits multiple haplotype markers rather than the 2-haplotype markers even the 2-haplotype markers were many more than multi-haplotype markers, and so was for the candidate gene-alleles (Supplementary Tables S1, S4). Thus we believe multiple haplotypes/alleles in a bi-phenotypic trait is a popular phenomenon.

This phenomenon in fact started with WA in which five haplotypes existed already for PC in the history even with only one phenotype. That means a single color conferred by five haplotypes is an existed fact and that five haplotypes may confer a same phenotype in Mendelian inheritance system, the same situation is for FC. After domestication in LR and RC, a same haplotype associated with two phenotypes is also a popular phenomenon, among the 11 haplotypes, ten associated with two phenotypes, while this phenomenon happened even in WA; it happened not only to the old haplotypes, but also to the new haplotypes, such as *PC-1-2* is a new haplotype in LR (Table 2). Among the 11 haplotypes on two loci, only one *FC-1*-5 confers one phenotype. The similar phenomenon also happened to the gene-alleles on the two pigmentation genes, 11 of the 12 alleles confer two or more phenotypes, *a7* for PC is newly emerged allele in RC, *a8* and *a9* are new alleles in LR, but the latter two alleles conferring only one phenotype in LR compared with WA (Table 2), totally three (*a8*, *a9*, and *b3*) out of the 12 alleles conferred a single phenotype.

### Marker-Haplotype and Candidate Gene-Allele Structure Changes of Bi-Phenotypic Traits in the Evolution From WA to LR Then to RC

For *PC-1* of WA, five of the six haplotypes existed while *PC-1-3* was the major one; in LR, six haplotypes existed, *PC-1-2* emerged, and *PC-1-1* and *PC-1-3* became the major ones; in RC, six haplotypes existed, both *PC-1-1* and *PC-1-2* are the major ones (Table 3). For *FC-1* of WA, five haplotypes existed, *FC-1-3*, *FC-1-4*, and *FC-1-5* were the major ones; in LR, all five haplotypes exist, but *FC-1-1* and *FC-1-2* became the major ones; in RC, only three haplotypes existed while *FC-1-4* and *FC-1-5* were absent (Table 3). Thus from WA to LR, *PC-1-2* emerged, *PC-1-1*, *FC-1-1* and *FC-1-2* became major ones; from LR to RC, the three major ones in LR continue as the major ones plus the one emerged in LR became also the major ones, but two haplotypes excluded and two haplotypes with frequency even less than 1.0% in RC. That means from WA to LR and RC, their population genetic structure, especially the major haplotypes has changed obviously along with some haplotypes emerged and some excluded due to the domestication and artificial selection.

**TABLE 3:** Frequency distribution of marker-haplotypes/gene-allele show the evolutionary relationship among populations/ecoregion subpopulations (%).

Locus/gene	Haplotype/Allele	WA	WA_*I*_	WA_*II*_	WA_*III*_	WA_*IV*_	LR	LR_*I*_	LR_*II*_	LR_*III*_	LR_*IV*_	RC	RC_*I*_	RC_*II*_	RC_*III*_	RC_*IV*_
*PC-1*	*PC-1-1*^*a*^	0.6^*b*^	−	−	−	4.2	42.4	31.1	43.6	59.0	36.8	37.9	4.5	42.1	61.3	69.6
	*PC-1-2*	−	−	−	−	−	11.9	31.1	10.0	9.0	9.2	49.1	89.4	38.8	27.4	18.8
	*PC-1-3*	58.8	41.8	57.6	79.5	62.5	20.7	11.1	9.1	12.8	35.0	1.1	−	1.1	1.6	2.9
	*PC-1-4*	17.6	32.7	16.9	9.1	−	14.4	22.2	34.5	7.7	1.8	9.2	2.3	16.4	8.1	4.3
	*PC-1-5*	15.9	23.6	13.6	2.3	29.2	9.1	4.4	2.7	11.5	13.5	2.0	2.3	1.1	1.6	4.3
	*PC-1-6*	7.1	1.8	11.9	9.1	4.2	1.5	−	−	−	3.7	0.7	1.5	0.5	−	−
*FC-1*	*FC-1-1*	6.6	−	5.1	13.6	12.5	62.4	62.2	42.7	78.2	68.1	48.2	38.6	55.7	41.9	52.2
	*FC-1-2*	2.7	3.6	1.7	4.5	−	31.8	33.3	46.4	19.2	27.6	51.6	60.6	44.3	58.1	47.8
	*FC-1-3*	34.6	18.2	20.3	52.3	75.0	1.8	−	0.9	1.3	3.1	0.2	0.8	−	−	−
	*FC-1-4*	26.4	25.5	47.5	11.4	4.2	3.3	4.4	10.0	−	−	−	−	−	−	−
	*FC-1-5*	29.7	52.7	25.4	18.2	8.3	0.8	−	−	1.3	1.2	−	−	−	−	−
*Glyma06g21551*	*a1*.CTATA^*a*^	79.7^*b*^	90.9	72.9	77.3	75.0	77.8	64.4	83.6	85.9	73.6	49.6	9.1	60.1	71.0	79.7
	*a2*.TCAAC	6.6	1.8	10.2	9.1	4.2	13.1	31.1	9.1	9.0	12.9	48.9	90.9	38.3	25.8	17.4
	*a3*.CTTTA	11.5	5.5	15.3	13.6	12.5	7.8	4.4	4.5	3.8	12.9	0.4	−	−	1.6	1.4
	*a4*.CCAAC	1.1	−	−	−	8.3	0.5	−	−	1.3	0.6	0.4	−	0.5	−	1.4
	*a5*.CCATA	0.5	1.8	−	−	−	0.3	−	0.9	−	−	−	−	−	−	−
	*a6*.TCAAA	0.5	−	1.7	−	−	−	−	−	−	−	0.2	−	−	1.6	−
	*a7*.TCATA	−	−	−	−	−	−	−	−	−	−	0.4	−	1.1	−	−
	*a8*.CTAAC	−	−	−	−	−	0.3	−	0.9	−	−	−	−	−	−	−
	*a9*.TTAAC	−	−	−	−	−	0.3	−	0.9	−	−	−	−	−	−	−
*Glyma13g04210*	*b1*.GG	88.5	100.0	94.9	70.5	79.2	61.4	57.8	70.9	55.1	58.9	78.7	94.7	71.6	74.2	71.0
	*b2*.AG	5.5	−	3.4	9.1	16.7	38.4	42.2	29.1	44.9	40.5	21.3	5.3	28.4	25.8	29.0
	*b3*.GA	6.0	−	1.7	20.5	4.2	0.3	−	−	−	0.6	−	−	−	−	−

*WA, annual wild population; LR, farmers’ landrace population; and RC, released cultivar population. I: Northern Single Cropping, Spring Planting Ecoregion; II: Huang-Huai-Hai Double Cropping, Spring and Summer Planting Ecoregion; III: Middle and Lower Changjiang Valley Double Cropping, Spring and Summer Planting Ecoregion; and IV: South and Southwest China Multiple Cropping Multiple Planting Ecoregion. PC, pubescence color; FC, flower color. *PC-1-1*: PC: pubescence color, -1: the first loci, -1: the first allele, the similar is for the others. ^*a*^The first haplotype/allele. ^*b*^The number is haplotype/allele frequency in population/ecoregion subpopulation (%).*

As for the population genetic structure changes in terms of candidate gene-alleles, the results were similar to those in associated SNPLDBs. For *Glyma06g21551* in WA, six of nine alleles existed with *a1* as the major allele and *a3* as the second major allele; in LR, seven alleles exist, with *a1* and *a2* as the major and second major alleles, and *a8* and *a9* newly emerged; in RC, six alleles existed with *a1* and *a2* as the major alleles, and *a5*, *a8* and *a9* excluded (Table 3). For *Glyma13g04210* in WA, three alleles existed with *b1* as the major allele; in LR, three alleles existed with *b2* and *b1* as the major ones; in RC, two alleles existed with *b2* and *b1* also as the major alleles, but *b3* absent (Table 3). Thus from WA to LR, *a8* and *a9* emerged, *b2* in addition to *b1* became the second major allele, and from LR to RC, *a5*, *a8*, *a9*, and *b3* excluded. That means from WA to LR and then to RC, their population genetic structure has changed obviously, some alleles emerged and some alleles excluded due to the domestication and artificial selection but the major alleles concentrated.

The haplotype/allele frequencies varied among populations or ecoregion subpopulations in WA, LR, and RC (Table 3). To trace the haplotypes/alleles back to WA, *PC-1-2* was absent in WA, but newly emerged in LR. This judgment was supported by that the binomial probability of its absence in WA in a large sample with 182 accessions due to sampling error is very small (much less than 1.00 × 10^–6^). The other four *PC-1* haplotypes/alleles can be traced to their wild ancestors. Among the QTL alleles (marker haplotypes), *PC-1-1* was from WA_*IV*_, *FC-1-1* can be traced to one of WA_*III*_, WA_*IV*_ and WA_*II*_, *FC-1-2* can be traced to one of WA_*III*_, WA_*II*_, and WA_*I*_. To trace the candidate gene alleles back to WA, *a4* was from WA_*IV*_, *a5* was from WA_*I*_, *b2*, and *b3* can be traced to one of WA_*III*_, WA_*IV*_, and WA_*II*_ (Table 3). To combine the above information, WA_*IV*_ involves two haplotypes/alleles and three candidate gene alleles traced from, WA_*III*_ involves two haplotypes and two candidate gene alleles traced from, WA_*II*_ involves two haplotypes/alleles and two candidate gene alleles traced from, and WA_*I*_ involves one haplotype/allele and one candidate gene allele traced from. In addition, the frequency of these haplotypes/alleles and candidate gene alleles in WA_*III*_ was higher than that in WA_*II*_ (Table 3). Thus WA_*IV*_ and WA_*III*_ have more original haplotypes/alleles and candidate gene alleles traced from, therefore, are more likely the wild sources of the cultivated soybeans. This concept supports the southern China origin hypothesis of cultivated soybean as reported by Gai et al. (2000) and Wen et al. (2009).

## Discussion

How to understand the association mapping results, are the multi-alleles conferring only two phenotypes exact and reasonable and is an allele conferring two phenotypes exact and reasonable which even with phenotypic ratio varying from allele to allele? These questions are to be answered. As indicated in the above discussion, we believe that both marker-haplotype and candidate gene-allele results are real facts. Thus we have to study further on how to understand and explain the facts. From our experiment, we suppose that in a natural population, mutation might cause multiple allele emergences, each emerged alleles may have phenotype different or similar to the old alleles. As we understand, the color phenotypes roughly were classified into two categories, but may varying in color darkness which might involving multiple alleles, this is one possible reason (Palmer et al., 2004). But the followed question is that what is the mechanism of a same gene-allele conferring two different phenotypes and different alleles have different ratios between the two phenotypes? As we considered, one possible cause might be the DNA sequence variation which was not detected in sequencing process or in other words, the hidden causal variation within a same gene-allele. In our study, we supposed all the haplotypes/alleles were grouped completely from our sequence data without intra-allele variation, but in fact the possibility of hidden causal variation within a SNPLDB-haplotype was not completely excluded because the sequencing depth not solid enough. Therefore, this possibility should be further checked. The other possible reason might be that the gene-allele was modified in their expressions or there might be some epigenetic reasons for the expression of individual alleles which is also to be explored.

Bolondi et al. (2017) reviewed the historical advances in gene concept, they discussed the two faced nature of genes, i.e., DNA phenotype and genotype, the DNA phenotype (the molecular constitution of the DNA and its epigenetic modifications) determines the coding information (the genotype), through gene expression the phenotype is obtained, following outer inputs the DNA genotype may influence the DNA phenotype. Many of the known epigenetic alterations are attributable to structural genetic variation acting, and thus are at least partly guided by DNA sequence (Hirsch et al., 2013). The functional gene state can be affected by physical modifications of a gene (e.g., methylation, epigenetics). This two-sided view of the gene allows to combine the genetic and epigenetic aspects in a unique solution, and may be able to explain the difference and similarity among the alleles on a locus (Hirsch et al., 2013). From the above, the simple inheritance trait might be not as simple as we thought before, especially in a natural population experienced a long term of historical period. It should be further explored for the genetic mechanisms.

## Data Availability Statement

The raw data supporting the conclusions of this article will be made available by the authors, without undue reservation, to any qualified researcher.

## Author Contributions

JG designed the research. FL performed the experiments. FL, JH, WW, and GX did the lab work. FL and JH analyzed the data. FL, JH, and JG drafted the manuscript.

## Conflict of Interest

The authors declare that the research was conducted in the absence of any commercial or financial relationships that could be construed as a potential conflict of interest.

## Footnotes

1 http://www.soybase.org
