# Supplementary material for: Bi-Phenotypic Trait May Be Conferred by Multiple Alleles in a Germplasm Population
Source: Front Genet. 2020 Jun 3;11:559. doi: 10.3389/fgene.2020.00559 (PMC7283545; doi:10.3389/fgene.2020.00559)
Supplement: Supplementary file 2 [file Data_Sheet_2.docx]

Supplementary Material

# Supplementary Tables

### Supplementary TABLE S1. Co-associated markers of the two Bi-phenotypic traits identified from χ^2^ association analysis and RTM-GWAS procedure.

| Locus | Marker | Haplotype  /allele | -log_10_p | | Locus | Marker | Haplotype  /allele | -log_10_p | *R*^2^ |
| --- | --- | --- | --- | --- | --- | --- | --- | --- | --- |
|  |  |  | PC/FC^a^ | Asso^b^ |  |  |  |  |  |
| *PC-1*^c^ | LDB_Gm06_17992357 | 6 | 85.9 | *-* | *PC-r-1*^d^ | LDB_Gm06_17992357(*PC-1*) | 6 | 53.2 | 25.3 |
| *PC-2* | LDB_Gm06_18122798 | 9 | 76.1 | >300.0 | *PC-r-2* | LDB_Gm03_47144971 | 4 | 26.8 | 11.7 |
| *PC-3* | LDB_Gm06_18725589 | 8 | 76.0 | 270.8 | *PC-r-3* | LDB_Gm06_18566657(*PC-5*) | 6 | 18.1 | 8.4 |
| *PC-4* | LDB_Gm06_17945997 | 4 | 74.3 | >300.0 | *PC-r-4* | LDB_Gm06_31287241 | 2 | 10.5 | 3.9 |
| *PC-5* | LDB_Gm06_18566657 | 6 | 71.5 | 221.5 | *PC-r-5* | LDB_Gm18_16251624 | 2 | 8.8 | 3.2 |
| *PC-6* | LDB_Gm06_17760273 | 9 | 67.9 | >300.0 | *PC-r-6* | LDB_Gm19_39952399 | 2 | 7.2 | 2.5 |
| *PC-7* | LDB_Gm06_18931498 | 7 | 61.7 | 244.7 | *PC-r-7* | LDB_Gm13_29926154 | 2 | 6.8 | 2.4 |
| *PC-8* | LDB_Gm06_18904014 | 6 | 60.1 | 209.8 | *PC-r-8* | LDB_Gm11_13236977 | 4 | 5.6 | 2.5 |
| *PC-9* | LDB_Gm08_8500850 | 4 | 58.4 | - | *PC-r-9* | LDB_Gm18_61308586 | 2 | 5.3 | 1.8 |
| *PC-10* | LDB_Gm06_18803176 | 2 | 51.7 | 125.7 | *FC-r-1* | LDB_Gm13_4369888(*FC-1*) | 5 | 289.6 | 69.1 |
| *FC-1* | LDB_Gm13_4369888 | 5 | 154.3 | - | *FC-r-2* | LDB_Gm13_4504313(*FC-8*) | 4 | 27.0 | 3.3 |
| *FC-2* | LDB_Gm13_4403078 | 6 | 152.2 | >300.0 | *FC-r-3* | LDB_Gm13_3496834(*FC-5*) | 7 | 26.3 | 3.5 |
| *FC-3* | LDB_Gm13_4357563 | 6 | 150.7 | >300.0 | *FC-r-4* | LDB_Gm13_4577385 | 2 | 9.2 | 0.9 |
| *FC-4* | LDB_Gm13_4257421 | 3 | 147.8 | 297.8 | *FC-r-5* | LDB_Gm13_4199950 | 3 | 9.3 | 1.0 |
| *FC-5* | LDB_Gm13_3496834 | 7 | 144.1 | >300.0 | *FC-r-6* | LDB_Gm13_4602511 | 6 | 8.1 | 1.1 |
| *FC-6* | LDB_Gm13_4299951 | 5 | 143.3 | >300.0 | *FC-r-7* | LDB_Gm13_3641866 | 5 | 7.0 | 0.9 |
| *FC-7* | LDB_Gm13_4504313 | 4 | 139.6 | >300.0 | *FC-r-8* | LDB_Gm14_444658 | 3 | 5.3 | 0.6 |
| *FC-8* | LDB_Gm13_4211768 | 4 | 135.2 | >300.0 |  |  |  |  |  |
| *FC-9* | LDB_Gm13_3674798 | 4 | 130.7 | >300.0 |  |  |  |  |  |
| *FC-10* | LDB_Gm13_3631255 | 2 | 130.6 | 158.6 |  |  |  |  |  |

Haplotype/allele: the number of haplotype/allele.

PC: pubescence color. FC: flower color.

^a^ PC/FC: associated trait.

^b^ Asso.: probability for the association between the locus and the first or most possible marker.

^c^ *PC-1*: the first associated locus of pubescence color by χ2 association analysis.

^d^ *PC-r-1*: the first associated locus of pubescence color by RTM-GWAS procedure.

### Supplementary TABLE S2. Associated and neighboring markers of PC identified from χ^2^ association analysis.

| Marker | Haplotype/Allele | -log_10_p | Physical position |  | Marker | Haplotype/Allele | -log_10_p | Physical position |
| --- | --- | --- | --- | --- | --- | --- | --- | --- |
| LDB_Gm06_17722798 | 2 | 4.08 | 17722798 |  | *PC-10*(LDB_Gm06_18803176) | 2 | 51.74 | 18803176 |
| LDB_Gm06_17741445 | 2 | 2.48 | 17741445 |  | LDB_Gm06_18803214 | 4 | 31.82 | 18803214 - 18826690 |
| *PC-6*(LDB_Gm06_17760273) | 9 | 67.91 | 17760273 - 17945810 |  | LDB_Gm06_18845190 | 2 | 1.14 | 18845190 |
| *PC-4*(LDB_Gm06_17945997) | 4 | 74.27 | 17945997 - 17980245 |  | LDB_Gm06_18894177 | 3 | 31.52 | 18894177 - 18899185 |
| LDB_Gm06_17980523 | 2 | 3.38 | 17980523 - 17992338 |  | LDB_Gm06_18899192 | 3 | 31.9 | 18899192 - 18902695 |
| *PC-1*(LDB_Gm06_17992357) | 6 | 85.93 | 17992357 - 18122264 |  | *PC-8*(LDB_Gm06_18904014) | 6 | 60.12 | 18904014 - 18931455 |
| LDB_Gm06_18122793 | 2 | 7.20 | 18122793 |  | *PC-7*(LDB_Gm06_18931498) | 7 | 61.72 | 18931498 - 18942913 |
| *PC-2*(LDB_Gm06_18122798) | 9 | 76.15 | 18122798 - 18288672 |  | LDB_Gm08_8346892 | 2 | 24.00 | 8346892 |
| LDB_Gm06_18326606 | 2 | 6.70 | 18326606 |  | LDB_Gm08_8371966 | 2 | 11.86 | 8371966 |
| LDB_Gm06_18353360 | 2 | 20.37 | 18353360 |  | LDB_Gm08_8444816 | 2 | 46.7 | 8444816 |
| LDB_Gm06_18356659 | 2 | 2.86 | 18356659 |  | LDB_Gm08_8469547 | 2 | 0.00 | 8469547 |
| LDB_Gm06_18356967 | 4 | 20.77 | 18356967 - 18483162 |  | LDB_Gm08_8471303 | 2 | 21.60 | 8471303 |
| LDB_Gm06_18483337 | 5 | 45.52 | 18483337 - 18502910 |  | *PC-9*(LDB_Gm08_8500850) | 4 | 58.39 | 8500850 - 8523539 |
| LDB_Gm06_18522337 | 2 | 3.32 | 18522337 - 18522350 |  | LDB_Gm08_8523560 | 3 | 40.63 | 8523560 - 8540446 |
| LDB_Gm06_18522475 | 2 | 2.66 | 18522475 |  | LDB_Gm08_8540484 | 2 | 1.39 | 8540484 |
| LDB_Gm06_18566597 | 2 | 3.27 | 18566597 |  | LDB_Gm08_8540508 | 2 | 3.67 | 8540508 |
| *PC-5*(LDB_Gm06_18566657) | 6 | 71.54 | 18566657 - 18704009 |  | LDB_Gm08_8540825 | 2 | 3.67 | 8540825 |
| LDB_Gm06_18717619 | 2 | 4.68 | 18717619 |  | LDB_Gm08_8555435 | 5 | 35.04 | 8555435 - 8640141 |
| *PC-3*(LDB_Gm06_18725589) | 8 | 75.97 | 18725589 - 18755723 |  |  |  |  |  |

PC: pubescence color.

Haplotype: the number of haplotype in a marker.

### Supplementary TABLE S3. Associated and neighboring markers of FC identified from χ^2^ association analysis.

| Marker | Haplotype/Allele | -log_10_p | Physical position |  | Marker | Haplotype/Allele | -log_10_p | Physical position |
| --- | --- | --- | --- | --- | --- | --- | --- | --- |
| *FC-5*(LDB_Gm13_3496834) | 7 | 144.10 | 3496834 - 3561958 |  | LDB_Gm13_4161386 | 2 | 6.30 | 4161386 |
| LDB_Gm13_3577726 | 2 | 17.16 | 3577726 - 3577900 |  | LDB_Gm13_4162607 | 5 | 18.95 | 4162607 - 4170948 |
| LDB_Gm13_3577927 | 2 | 3.00 | 3577927 |  | LDB_Gm13_4171075 | 2 | 3.30 | 4171075 |
| LDB_Gm13_3578611 | 2 | 0.00 | 3578611 |  | LDB_Gm13_4171435 | 2 | 3.30 | 4171435 |
| LDB_Gm13_3578708 | 4 | 23.69 | 3578708 - 3607916 |  | LDB_Gm13_4199950 | 3 | 96.91 | 4199950 - 4202043 |
| *FC-10*(LDB_Gm13_3631255) | 2 | 130.58 | 3631255 |  | LDB_Gm13_4202975 | 2 | 25.25 | 4202975 |
| LDB_Gm13_3641694 | 2 | 3.32 | 3641694 |  | *FC-7*(LDB_Gm13_4211768) | 4 | 135.20 | 4211768 - 4216313 |
| LDB_Gm13_3641866 | 5 | 125.25 | 3641866 - 3657693 |  | LDB_Gm13_4229679 | 2 | 7.22 | 4229679 |
| LDB_Gm13_3657994 | 2 | 3.43 | 3657994 |  | LDB_Gm13_4241986 | 2 | 6.17 | 4241986 |
| *FC-9*(LDB_Gm13_3674798) | 4 | 130.74 | 3674798 - 3749614 |  | LDB_Gm13_4242221 | 2 | 5.90 | 4242221 |
| LDB_Gm13_3769002 | 2 | 15.25 | 3769002 |  | *FC-4*(LDB_Gm13_4257421) | 3 | 147.80 | 4257421 - 4257442 |
| LDB_Gm13_3769077 | 2 | 6.96 | 3769077 |  | *FC-6*(LDB_Gm13_4299951) | 5 | 143.29 | 4299951 - 4357220 |
| LDB_Gm13_3769278 | 6 | 50.05 | 3769278 - 3790640 |  | LDB_Gm13_4357528 | 2 | 4.21 | 4357528 |
| LDB_Gm13_3790664 | 2 | 13.91 | 3790664 |  | *FC-3*(LDB_Gm13_4357563) | 6 | 150.72 | 4357563 - 4367722 |
| LDB_Gm13_3790672 | 2 | 4.08 | 3790672 |  | LDB_Gm13_4367731 | 2 | 7.39 | 4367731 |
| LDB_Gm13_3790961 | 2 | 4.21 | 3790961 |  | LDB_Gm13_4369601 | 2 | 3.17 | 4369601 |
| LDB_Gm13_3791021 | 2 | 5.64 | 3791021 |  | *FC-1*(LDB_Gm13_4369888) | 5 | 154.32 | 4369888 - 4402736 |
| LDB_Gm13_3791028 | 2 | 3.57 | 3791028 |  | *FC-2*(LDB_Gm13_4403078) | 6 | 152.24 | 4403078 - 4472990 |
| LDB_Gm13_3811058 | 6 | 51.87 | 3811058 - 3861120 |  | LDB_Gm13_4479350 | 2 | 1.87 | 4479350 |
| LDB_Gm13_3928439 | 3 | 27.59 | 3928439 - 3930937 |  | LDB_Gm13_4484118 | 2 | 45.01 | 4484118 |
| LDB_Gm13_3931134 | 5 | 11.81 | 3931134 - 4020449 |  | LDB_Gm13_4496850 | 2 | 0.33 | 4496850 |
| LDB_Gm13_4036639 | 2 | 0.00 | 4036639 |  | LDB_Gm13_4497150 | 2 | 39.42 | 4497150 |
| LDB_Gm13_4046998 | 2 | 2.13 | 4046998 |  | LDB_Gm13_4497187 | 3 | 40.90 | 4497187 - 4497868 |
| LDB_Gm13_4103535 | 2 | 2.39 | 4103535 |  | LDB_Gm13_4497895 | 2 | 32.53 | 4497895 |
| LDB_Gm13_4103555 | 5 | 27.43 | 4103555 - 4155439 |  | LDB_Gm13_4497910 | 3 | 55.92 | 4497910 - 4498244 |
| LDB_Gm13_4160993 | 2 | 11.17 | 4160993 |  | LDB_Gm13_4498257 | 2 | 2.00 | 4498257 |
| LDB_Gm13_4161003 | 2 | 14.39 | 4161003 |  | *FC-8*(LDB_Gm13_4504313) | 4 | 139.58 | 4504313 - 4550943 |
| LDB_Gm13_4161034 | 2 | 2.52 | 4161034 |  |  |  |  |  |

FC: Flower color.

Haplotype: the number of haplotype in marker.

### Supplementary TABLE S4. The candidate gene associated with the bi-phenotypic trait in CSGP.

| Locus | Candidate gene | SNP | P value | Allele | Annotation |
| --- | --- | --- | --- | --- | --- |
| *PC-1* | *Glyma06g21551* | Gm06_18121962 | 6.1E-216 | *a1*.CTATA^a^ | Beta-Glucuronidase |
|  |  | Gm06_18122264 | 7.9E-211 | *a2*.TCAAC |  |
|  |  | Gm06_18122793 | 2.4E-19 | *a3*.CTTTA |  |
|  |  | Gm06_18122798 | 6.6E-213 | *a4.*CCAAC |  |
|  |  | Gm06_18123131 | 6.8E-211 | *a5*.CCATA |  |
|  |  |  |  | *a6*.TCAAA |  |
|  |  |  |  | *a7*.TCATA |  |
|  |  |  |  | *a8*.CTAAC |  |
|  |  |  |  | *a9*.TTAAC |  |
| *FC-1* | *Glyma13g04210* | Gm13_4552834 | 3.5E-74 | *b1*.GG | Flavonoid 3'-monooxygenase activity |
|  |  | Gm13_4552847 | 1.6E-28 | *b2*.AG |  |
|  |  |  |  | b3.GA |  |

PC: pubescence color; FC: flower color.

*P* value: the included SNPs in genes were tested for their significant association to the locus by the χ^2^ analysis.

^a^ The first allele.

The candidate genes around the associated locus (±100 Kb) were obtained based on the soybean genetics and genomics database. The gene calls and annotations were retrieved from *G. max* (version Wm82.a1.v1.1) in SoyBase (<http://www.soybase.org>).

## Supplementary Figure


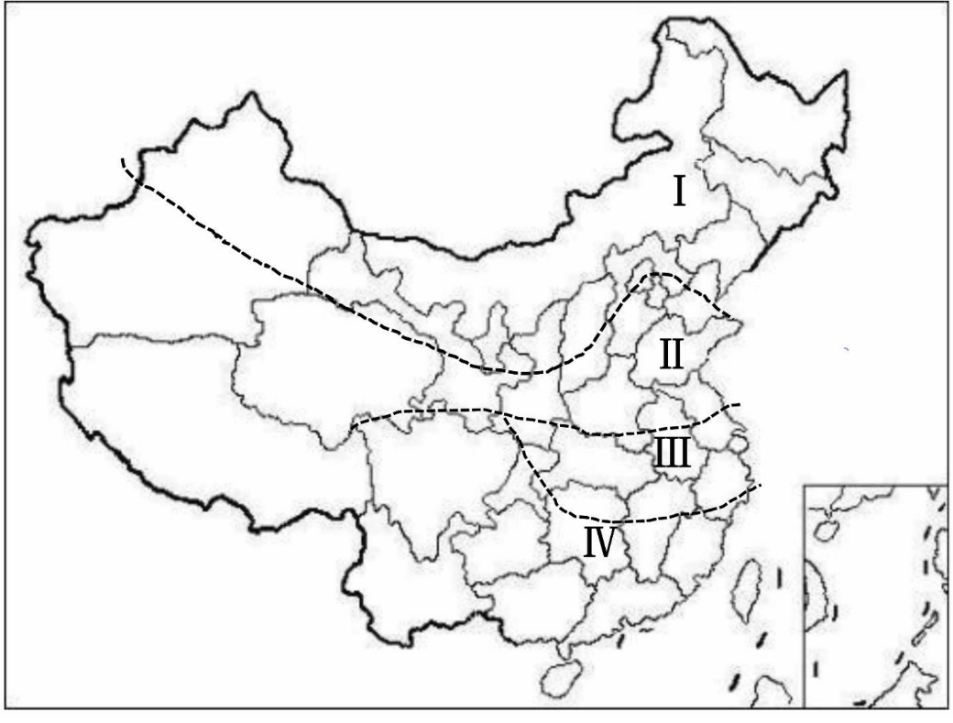


### Supplementary FIGURE S1. Soybean ecoregions in China

I. Northern Single Cropping, Spring Planting Ecoregion; II: Huang-Huai-Hai Double Cropping, Spring and Summer Planting Ecoregion; III: Middle and Lower Changjiang Valley Double Cropping, Spring and Summer Planting Ecoregion; IV: South and Southwest China Multiple Cropping Multiple Planting Ecoregion.
